# Risk-of-Malignancy Index in preoperative evaluation of clinically restricted ovarian cancer

**DOI:** 10.1590/S1516-31802002000300003

**Published:** 2002-05-02

**Authors:** José Carlos Campos Torres, Sophie Françoise Mauricette Derchain, Aníbal Faúndes, Renata Clementino Gontijo, Edson Zangiacomi Martinez, Liliana Aparecida Lucci de Ângelo Andrade

**Keywords:** Ovarian cancer, CA 125, Ultrasound, Menopausal status, Risk of malignancy, Câncer de ovário, CA 125, Ultra-sonografia, Estado menopausal, Risco de malignidade

## Abstract

**CONTEXT:**

There is no adequate preoperative method for differentiating between benign and malignant pelvic masses. Evaluations of CA 125 serum levels, ultrasonography findings and menstrual state have been tested in isolation as diagnostic methods. The evaluation of these three methods in association with each other could improve diagnostic performance.

**OBJECTIVE:**

To evaluate the risk-of-malignancy index by combining serum CA 125 levels, ultrasound score and menopausal status in preoperative diagnoses for women with pelvic masses clinically restricted to the ovaries and without clear evidence of malignancy.

**DESIGN:**

Cross-sectional study.

**SETTING:**

Centro de Atenção Integral à Saúde da Mulher, Universidade Estadual de Campinas, Campinas, São Paulo, Brazil.

**PARTICIPANTS:**

158 women admitted between January 1996 and March 1998 for surgical exploration of pelvic masses.

**PROCEDURES:**

The risk-of-malignancy index was calculated as US x M x CA 125, performed preoperatively. Ultra-sound findings were classified according to the shape, size, multiplicity, presence of wall expansion involvement or ascites, using a score system (US). Menopausal status was considered as 1 for premenopausal and 3 for postmenopausal (M), and CA 125 serum levels were considered in absolute values.

**STATISTICAL ANALYSIS:**

Most relevant variables were included in a logistic multiple regression model, fitted using the ultrasound score, the serum CA 125 level and the menopausal status. The model was used for evaluating the performance of each individual predictor in determining the malignancy of these tumors and identifying the risk-of-malignancy index.

**RESULTS:**

The best individual performance was found in CA 125 levels (sensitivity of 78%, specificity of 75%), followed by ultrasound score (sensitivity of 75%, specificity of 73%) and menopausal status (sensitivity of 73%, specificity of 69%). The performance obtained for the risk-of-malignancy index at the cut-off point of 150 was a sensitivity and specificity of 79%. The area under the ROC curve for the risk-of-malignancy index was 0.90, which was greater than the area for CA 125 levels (0.83) or ultrasound score (0.79).

**CONCLUSION:**

The risk-of-malignancy index using ultrasound morphological score, serum CA 125 levels and menopausal status might be of value in the preoperative assessment of ovarian carcinomas.

## RESUMO

**CONTEXTO:**

Não existem métodos pré-operatórios adequados para diferenciar massas pélvicas benignas de malignas. A avaliação do CA 125, dos achados ultra-sonográficos e do estado menstrual têm sido testados como métodos diagnósticos isolados. O uso destes três métodos em associação poderia levar a um aumento do seu desempenho.

**OBJETIVOS:**

Avaliar o índice de risco de malignidade incorporando a dosagem de CA 125 sérico, escore ultra-sonográfico e estado menopausal, no diagnóstico pré-operatório de mulheres com massas pélvicas clinicamente restritas ao ovário e sem evidências claras de malignidade.

**TIPO DE ESTUDO:**

Estudo de corte transversal.

**PARTICIPANTES:**

158 mulheres admitidas entre janeiro de 1996 à março de 1998 para exploração cirúrgica de massa pélvica ovariana.

**PROCEDIMENTOS:**

O índice de risco de malignidade foi calculado como US x M x CA 125, avaliados pré-operatoriamente. Achados ultra-sonográficos foram classificados de acordo com a forma, tamanho, multiplicidade, presença de envolvimento de parede ou ascite em um sistema de escore (US). Para o estado menopausal foi considerada a pontuação de 1 para pré-menopausal e 3 para pós-menopausal (M), e a dosagem dos níveis séricos de CA 125 sérico foi considerada em seu valor absoluto.

**ANÁLISE ESTATÍSTICA:**

As variáveis mais relevantes foram incluídas em um modelo de regressão logística múltipla, usando o escore ultra-sonográfico, o nível de CA 125 e o estado menopausal. Foi usada para avaliar o desempenho de cada preditor individual em determinar a malignidade destes tumores e identificar o índice de risco de malignidade.

**RESULTADOS:**

O melhor desempenho individual foi encontrado na dosagem de CA 125 (sensibilidade de 78%, especificidade de 75%), seguido pelo escore ultra-sonográfico (sensibili-dade de 75%, especificidade de 73%) e estado menopausal (sensibilidade de 73%, especificidade de 69%). O desempenho obtido pelo índice de risco de malignidade no ponto de corte de 150 foi sensibilidade e especificidade de 79%. A área sob a curva ROC para o índice de risco de malignidade foi 0,90, maior que a área para o CA 125 (0,83) ou escore ultra-sonográfico (0,79).

**CONCLUSÃO:**

O índice de risco de malignidade usando escore morfológico ultra-sonográfico, dosagem de CA 125 sérico e estado menopausal pode ser de valor no acesso pré-operatório do carcinoma ovariano.

## INTRODUCTION

Ovarian cancer remains the third most frequent gynecological neoplasm and corresponds to the highest mortality rate in developed countries.^1^ In Brazil, according to Datasus files, the incidence of malignant ovarian tumors was reported to be 3.6 per 100,000 women in 1998, resulting in 1830 deaths in the same year.^2^ A worse prognosis is correlated with late diagnosis. Up to 70% of the cases are detected at advanced stages, with increased ovarian disease, in which the mortality rate reaches 70% within two years and 90% within five years, which has encouraged research into ovarian cancer screening methods.^1,3,4^ However, these are costly methods and, because of their elevated false-positive results, they have been ineffective.^5^ Ovarian tumors are presented as adnexal masses which give rise to a number of different benign and malignant conditions. The accurate diagnosis of an adnexal mass is a challenge for the gynecologist, because of its bizarre and atypical behaviour.^6,7^ Preoperative diagnostic procedures that are able to distinguish whether an ovarian neoplasm is malignant or benign, could be useful in planning optimized treatment. Until now, the standard strategy for differential diagnosis has been exploratory laparotomy. On the other hand, detailed analysis of the origin of the pelvic mass has encouraged the use of minimal invasive surgery, such as laparoscopy or mini-laparotomy, in selected cases.^8-10^ A preoperative suggestion of malignancy can guide the gynecologist to refer women with suspected pelvic masses to an oncological unit for appropriate therapy and optimized debulking.^6,7,11^

Several diagnostic methods for pelvic masses have been reported, such as abdominal and transvaginal ultrasonography, three-dimensional ultrasound, color Doppler ultrasonography and tumor markers.^12,13^ However, none of these methods used individually has shown significantly better performance in detecting malignant tumors from clinically restricted ovarian masses. The development of a mathematical formula using a logistic model, incorporating menopausal status, the serum level of a glycoprotein called CA 125 (which is considered to be a tumor marker) and ultrasound findings in a score system, has been described in the literature in the form of different malignancy indexes. These indexes were calculated using a simplified regression equation obtained from the product of the ultrasound findings score, the menopausal status score and the absolute value of CA 125 serum levels. Jacobs et al. originally developed the risk-of-malignancy index in 1990 and it is termed the risk-of-malignancy index #1. Tingulstad et al. developed a risk-ofmalignancy index in 1996, known as risk-ofmalignancy index #2 and in 1999 they modified it to form the risk-of-malignancy index #3. The difference between the three indices lies in the different scorings of ultrasound findings and menopausal status.^14-16^ All indices presented a significantly better performance in diagnosing malignancy than did each predictor taken separately. These indices were tested by Morgante et al.^18^ on another population with evident malignant criteria in the ultrasonography, such as hepatic or distant metastasis, and they found that the risk-of-malignancy index #2 performed better for detecting ovarian malignancy.

Previous studies did not show the usefulness of the score among women with lesions clinically restricted to the ovaries and without clear evidence of malignancy. The purpose of this study was to evaluate the risk-of-malignancy index combining serum CA 125 levels, ultrasound score and menopausal status, in the preoperative diagnosis for women with pelvic masses clinically restricted to the ovaries and without clear evidence of malignancy.

## METHOD

Women with a pelvic mass apparently restricted to the adnexal region who had appointments for laparotomy at the Centro de Atenção Integral à Saúde da Mulher, Universidade Estadual de Campinas were selected. Between January 1996 and March 1998, 158 women were included in the study after signing a consent form approved by the Research Ethics Committee of the University. Twenty-one patients with evident signs of hepatic and intraperitoneal metastasis and six with lung metastasis were excluded. The CA 125 serum levels, ultrasound findings and menopausal status were registered preoperatively.

Serum CA 125 samples were assayed by radioimmunoassay (Malvern, Pennsylvania, USA). The ultrasound examination was performed using a 3.75-MHz abdominal convex transducer (TOSHIBA SSA-140, Japan, and ACUSON XP4A, USA). Women with tumors bigger than 10 cm underwent transvaginal scanning with a 7.5-MHz transducer. The lesions were evaluated according to the shape, size, multiplicity and presence of wall expansion involvement or ascites. Morphological evaluation was performed using the inner wall structure, wall thickness, presence of septa and their thickness and echogenicity.^18,19^ Six levels of increasing malignancy and two levels of associated lesions were defined. Using logistic regression, a score was attributed to each ultrasound finding, termed the ultrasound score. Postmenopausal status was defined as more than one year of amenorrhea or an age of more than 50 years in women who had had a hysterectomy. All other women were considered premenopausal. Women were submitted to laparotomy, and the tissue excised was sent for histopathological analysis. Histopathological diagnosis was considered as the gold standard for defining the outcome and it was classified as benign or malignant.^20^

The diagnostic ability of each variable was evaluated in a univariate analysis, using the odds ratio as an associated measure. Most relevant variables were included in a logistic multiple regression model, fitted using the ultrasound findings defined above, and the serum CA 125 level and menopausal status. This risk-of-malignancy index was calculated with the attribution of values of 1 for premenopausal status and 3 for postmenopausal status (M), versus ultrasound score (US) and the absolute values of CA 125 serum levels: US x M x CA 125 (Table 1).

**Table 1:** Risk-of-malignancy index according to ultrasound findings, absolute values of CA 125 serum levels and menopausal status

Ultrasound findings		Score
Unilocular simple cysts with regular fine wall or lesion suggesting dermoid cyst.		0
Multilocular cyst with regular and smooth wall (<3 mm) or thick (>3 mm) or solid homogeneous tumor with hyperechogenic and well-defined wall.		1
Unilocular cyst or multilocular cyst with fine wall, with irregularity in the wall or septa (>3 mm).		2
Multilocular cyst with thick and irregular wall (irregularity <3 mm), and/or irregular septa; or cyst with papillary irregularity over 3 mm.		4
Complex lesion, with predominance of cystic or solid area, without irregularity in surface.		5
Complex lesion with irregularity in surface (<3 mm) or badly-defined and irregular wall; or solid heterogeneous lesion.		10
Multiplicity - Unilateral lesion or bilateral lesions.		0
Associated lesions:	ascites	1
	wall expansive involvement	2
	greater than 3 mm	
Ca 125 serum levels		0 - ∞
Premenopausal		1
Postmenopausal		3

Sensitivity, specificity and likelihood ratio were calculated for different cut-off points of CA 125, ultrasound score and the resultant risk-of-malignancy index. Empirical receiver operating characteristic curves were used for showing the overall diagnostic ability of serum CA 125, ultrasound score and the risk-ofmalignancy index. All statistical analyses was done using the SAS software, version 8.0.

## RESULTS

According to the histological examination of the surgical specimens of the 158 women, 67 (42.4%) had malignant and 91 (57.6%) had benign disease. The majority of the women with malignant disease had ovarian cancer; one had a Kruckenberg gall bladder tumor and three had non-ovarian gynecological neoplasia. The ovarian cancers included 37 at FIGO^11^ stage I, two at stage II and 19 at stage IIIc of the disease. Among women with stage III disease, 15 (79%) presented only lymph node invasion (Table 2).

**Table 2:** Distribution of diagnoses and the International Federation of Gynecology and Obstetrics Stages

HISTOLOGICAL FINDINGS	N
Ovarian carcinoma	
Stage I borderline tumors	18
Stage I invasive tumors	19
Stage II	2
Stage IIIa	1
Stage lllc (peritoneal masses)	4
Stage lllc (lymph node)	15
Germ cells tumor	2
Ovarian carcinosarcoma	1
Ovarian sarcoma	1
Leiomyosarcoma	3
Kruckenberg tumor	1
**Total maliqnant cases**	**67**
Ovarian serous epithelial cysts	18
Ovarian mucinous epithelial cysts	12
Follicular cysts	7
Non ovarian cysts	9
Ovarian fibroma	14
Leiomyoma	4
Dermoid cysts	19
Pelvic inflammatory disease	3
Endometriosis	4
Genital tuberculosis	1
**Total benign cases**	**91**

**Figure 1:**
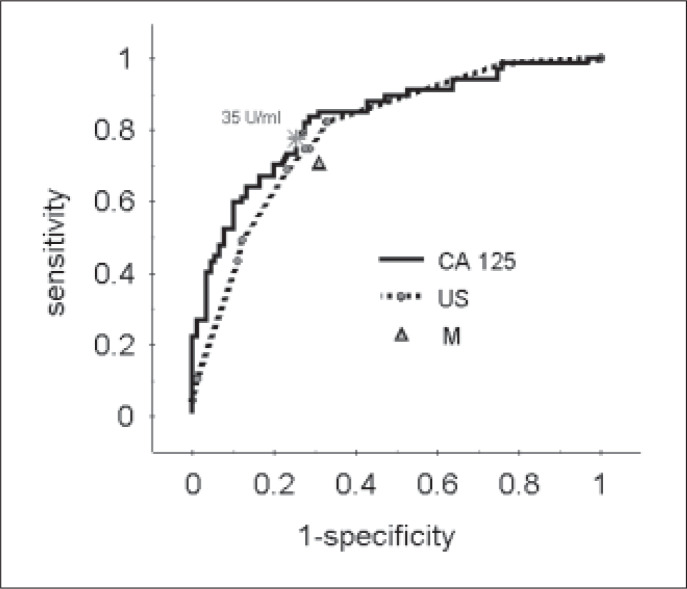
Receiver operating characteristic curves of the individual predictors showing the relationship between sensitivity and specificity of CA 125 serum level, ultrasound score (US) and menopausal status (M) in the discrimination between benign and malignant pelvic masses.

**Figure 2:**
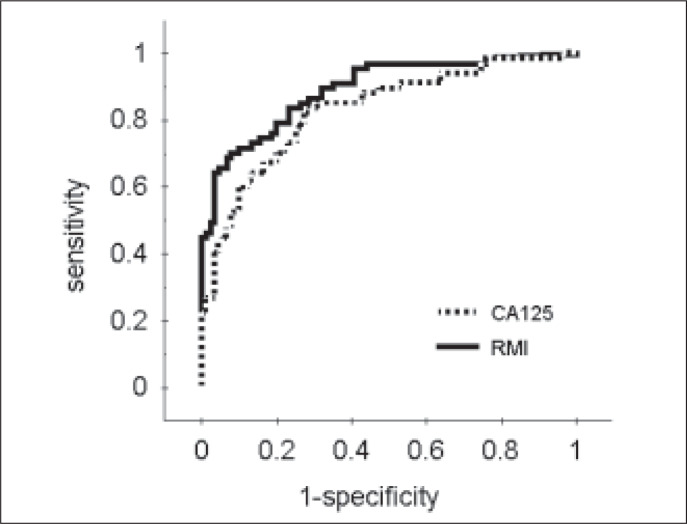
Receiver operating characteristic curves for the risk-of-malignancy index (RMI) and CA 125 levels in the discrimination between benign and malignant pelvic masses.

The sensitivity, specificity and positive and negative likelihood ratios of serum CA 125, ultrasound score and menopausal status are reported in Table 3. The performance obtained for a serum CA 125 level of 35 U/ml was a sensitivity of 78% and specificity of 75%. The sensitivity and the specificity of ultrasound scores of three or four were 75% and 71%, respectively. In the receiver operating characteristic curve evaluation, just as in the logistic regression analysis, CA 125, ultrasound score and menopausal status were found to be relevant predictors of malignancy. The area under the receiver operating characteristic curve for CA 125 was 0.83 and for the ultrasound score was 0.79 (Figure 1). Sensitivity, specificity and likelihood ratios of the risk-of-malignancy index at different cutoff points are also showed in Table 3. The confidence interval for the risk-of-malignancy index was not estimated, because the receiver operating characteristic curve variability associated with this index could only be evaluated using a second validation sample. The performance obtained for the risk-ofmalignancy index at the cut-off point of 150 was a sensitivity and specificity of 79%. The area under the receiver operating characteristic curve for the risk-of-malignancy index was 0.90, which was more than the area for the CA 125 serum levels (Figure 2).

**Table 3:** Sensitivity, specificity and likelihood ratios for predicting malignancy of CA 125 serum levels, ultrasound score, menopausal status and the risk-of-malignancy index

Variables	Sensitivity (%)	95% CI	Sensitivity (%)	95% CI	Likelihood Ratio (+)	Likelihood Ratio (-)
CA 125 (u/ml) ^*^						
10	94	(84 - 98)	26	(17 - 37)	1.27	0.23
35	78	(65 - 86)	75	(64 - 83)	3.12	0.29
50	61	(48 - 73)	88	(78 - 94)	5.08	0.44
65	52	(39 - 64)	91	(82 - 96)	5.77	0.53
150	27	(17 - 39)	97	(89 - 99)	9.00	0.75
Ultrasound Score ^*^						
1	98	(90 - 99)	24	(16 - 35)	1.29	0.08
2	82	(70 - 90)	67	(56 - 76)	2.48	0.27
3	75	(62 - 84)	71	(60 - 80)	2.59	0.35
4	75	(62 - 84)	73	(62 - 81)	2.78	0.34
8	43	(31 - 56)	89	(80 - 94)	3.91	0.64
Postmenopausal						
Status	73	(60-83)	69	(58-78)	2.35	0.39
Risk of malignancy index ^*^						
30	96		56		2.18	0.07
100	84		77		3.65	0.21
150	79		79		3.76	0.27
200	73		86		5.21	0.31
500	63		97		21.00	0.38

* *Cut-off point;*

*CI: Confidence Interval.*

## DISCUSSION

In women without evidence of advanced-stage ovarian cancer, the current risk-ofmalignancy index is useful in clinical practice for differentiating malignant from benign pelvic masses, as compared to each individual component measured separately. In the present population, this index was more accurate in comparison with the best individual predictor and CA 125 serum level. No increase in the accuracy was observed when analyzing patients' ages, tumor measurements or bilaterally. The validity of the index depends on the proportions of malignant neoplasm and benign processes and the proportions of initial and advanced stages.^21,22^

In both studies carried out by Jacobs et al.14 and Tingulstad et al.,^15,16^ a non-selected population was used that included patients with systemic metastases. At a cut-off point of 200, Jacobs et al. found a sensitivity of 73% and a specificity of 91%. Tingulstad et al. found a sensitivity of 76% and specificity of 82% in 1996, and 74% and 91% respectively in 1999. The index showed itself useful in referring patients with advanced neoplasia to a more complex healthcare unit. They reported that 22% of the cases were in stage I and 35% in II. Although the previous indices were applied to cases in more advanced stages, quite possibly the best application for the index could be for those cases without ultrasonographic evidence of malignancy. This occurs because the risk-of-malignancy index system translates the morphological description of pelvic mass into objective numerical data, reducing the bias attributable to the examiner's subjectivity. The recent development of ultrasound techniques and the better characterization of malignant masses by this method have led to better performance by ultrasound as a predictor of malignancy, especially in those cases with hepatic, intraabdominal or neighboring organ metastases.^13^ Ascites associated with pelvic masses is a recognized sign of malignancy.^23^ Some cases of rare non-neoplastic conditions are also associated with ascites. Despite this, no association was found between ascites and malignancy in this study, in a univariate analysis (data not shown).

In the present study, the malignant ovarian neoplasm group consisted mainly of early invasive or borderline tumors (67%). Among the advanced tumors, the majority of the stage III cases were so classified due to lymph node invasion. None of the cases presented clear preoperative evidence of metastasis. Borderline tumors and benign processes can be treated in a general hospital by gynecologists, although invasive neoplasia, particularly advanced invasive cases, merits appropriate therapy by highly skilled surgical teams in specific oncology centers. The risk-of-malignancy index facilitates the selection of cases for referral to an oncological unit and also helps the surgeon to choose the surgical approach. For example: a premenopausal 35-year-old woman with a solid well-defined wall tumor and a CA 125 serum level of 45 U/ml presents a risk-of-malignancy index of 45. At the cut-off point of 150, the tumor can be considered as benign and the false-negative probability is around 21%. However, the probability at the cut-off point of 100 is 16%. A tumor with the same characteristics, in a postmenopausal 65-year-old woman, with a CA 125 level of 97 U/ml has a risk-of-malignancy index of 291. At the cutoff point of 150, this tumor can be considered as malignant with a false-positive probability of around 21%, and at the cut-off point of 200 this tumor is still considered as malignant, with a false-positive probability of 14%.

## CONCLUSION

In conclusion, the risk-of- malignancy index is apparently able to identify the probability of malignant pelvic masses, by incorporating serum CA 125 serum levels, ultrasound morphology and menopausal status, performed individually in women with ovarian masses. The main purpose of this study was the evaluation of a risk-of-malignancy index defined in a selected population of apparently early lesions. This index is a simple score system which can be applied directly to clinical practice and might be of value in the preoperative assessment of the adnexal mass. It showed itself useful in referring patients with advanced neoplasia to a more complex healthcare unit, although it does not seem to show prognostic value. However, the performance of the present index must be evaluated in other studies, using a validation sample from a similar population.
